# Nanotechnology in brain cancer treatment: The role of gold nanoparticles as therapeutic enhancers

**DOI:** 10.1002/ibra.12198

**Published:** 2025-05-10

**Authors:** Simona Tarantino, Annalisa Bianco, Valeria De Matteis, Edoardo Scarpa, Rosaria Rinaldi

**Affiliations:** ^1^ Department of Mathematics and Physics “Ennio De Giorgi” University of Salento Via Arnesano Lecce Italy; ^2^ Department of Experimental Medicine ‐ University of Salento Centro Ecotekne Monteroni Lecce Italy; ^3^ Institute for Microelectronics and Microsystems (IMM) National Research Council (CNR) Via Monteroni Lecce Italy; ^4^ Department of Pharmaceutical Sciences University of Milan Milan Italy; ^5^ National Institute of Molecular Genetic (INGM) Milano Italy

**Keywords:** brain, brain cancer, gold nanoparticles, nanotechnology, treatment

## Abstract

Brain cancer, with glioblastoma (GBM) being one of the most aggressive and treatment‐resistant cancers, represents a leading cause of mortality and morbidity worldwide. Its complex nature and the presence of the blood‐brain barrier (BBB) significantly hinder the effectiveness of conventional therapies, posing major challenges for treatment development. In this context, nanotechnology—particularly nanomedicine—has emerged as a promising strategy to overcome these barriers and enhance standard treatments like chemotherapy and radiotherapy (RT). This review focuses on three of the most challenging brain neoplasms—GBM, brain metastases, and pediatric brain tumors—and explores the growing role of nanoparticle‐based therapies, with special emphasis on gold nanoparticles (AuNPs). Owing to their unique physicochemical properties, such as surface functionalization, biocompatibility, and the ability to cross the BBB, AuNPs have shown great potential in selectively delivering drugs, enhancing RT as radiosensitizers, and reducing systemic toxicity. Despite their therapeutic advantages, concerns remain regarding the long‐term safety of AuNPs. Their small size and ability to cross biological barriers may lead to unintended biodistribution, immune responses, and cytotoxic effects. Reported risks include inflammatory reactions, apoptosis, and developmental toxicity, highlighting the need for comprehensive safety assessments. AuNPs offer a promising avenue for improving therapeutic efficacy and patient survival in brain cancers. However, their clinical application requires further in‐depth preclinical and clinical evaluation to ensure both effectiveness and safety

## Graphical abstract

This review highlights the potential of gold nanoparticles (AuNPs) in overcoming the blood‐brain barrier (BBB) to improve brain tumor treatment. By enabling targeted drug delivery and enhancing therapies like radiotherapy, phototherapy, and chemotherapy, AuNPs offer a promising approach for safer, more effective treatments of glioblastoma and other brain cancers.

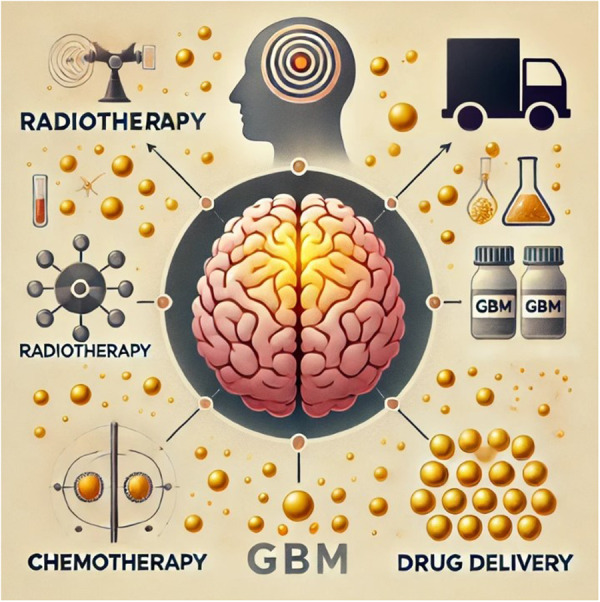

## 1 INTRODUCTION

Cancers of the central nervous system (CNS), classified as rare tumors, account for about 3% of all cancers worldwide, with a higher prevalence in men compared to women^1^, ^2^ They are recognized as some of the most fearsome forms of cancer due to the numerous challenges posed by the brain's intricate anatomy and physiology, which significantly complicate therapeutic treatment strategies.^3^ The brain's complex, three‐dimensional structure, with its dense network of neural tissue and vital structures, makes it difficult to access and completely excise tumors without risking significant neurological damage. Tumors located near critical areas such as the brainstem or motor cortex pose additional difficulties, as even minimal damage to these regions can result in permanent functional deficits. Furthermore, the anatomical variability between patients and the deep or diffuse nature of certain tumors can restrict both the precision and safety of surgical resection. The lack of clear boundaries between tumor and healthy tissue, combined with the potential for tumor recurrence, further complicates the surgical approach.^4^, ^5^ Additionally, the incidence of brain tumors appears to be rising, likely due to improvements in diagnostic techniques.^6^ Treating patients with these complex tumors remains a significative challenge in medical field, where early diagnosis and integrated care coordination are essential for patients outcomes.^7^ Currently, over 100 types of primary tumors of the CNS have been identified, each with distinct histological features, among which glioblastoma (GBM) ranks as the most prevalent primary malignant brain tumors (47.7%), with a 5‐year survival rate of 5.6%.^8^ The most frequent subtypes of glioma also include grade II‐III astrocytoma (23%), followed by oligodendroglioma and mixed glioma.^9^ Glioma incidence is higher in males, with diffuse astrocytoma and oligodendrogliomas most commonly affecting young adults, with median ages of 46 and 43 years, respectively. Survival rates for gliomas vary considerably, ranging from a 94.7% 5‐year survival for pilocytic astrocytoma to just 6.8% for GBMs. Additionally, about 5% of gliomas are familial, often associated with hereditary conditions such as Li‐Fraumeni syndrome, Turcot syndrome, and neurofibromatosis.^10^

The first critical aspect of such tumors is their location within brain, where effective surgical removal is particularly difficult.^11^ In fact, in addition to the brain's intricate anatomy and physiology, a key challenge in GBM surgery is its highly invasive nature, with tumors diffusely infiltrating surrounding tissue. These tumors diffusely penetrate the surrounding brain parenchyma, often without clear boundaries, which complicates efforts to achieve complete resection. The infiltrative cells, which are microscopically dispersed within healthy tissue, remain undetectable during surgery. Even small clusters of escaped tumor cells can repopulate the brain. This often leads to recurrence, which is highly resistant to conventional treatments such as radiation and chemotherapy. This ability of GBM cells to invade and adapt within the brain makes achieving long‐term remission particularly difficult.^12^ Another key factor hindering the treatment of CNS tumors is the blood‐brain barrier (BBB), placed between the blood and the cerebral interstitium, which works as an obstacle for drug delivery.^13^ Specifically, while BBB plays a crucial role in the bio‐functions of both maintaining the homeostasis and controlling the entry of molecules to the CNS area, it also restricts the ability of systemic chemotherapeutic agents to reach the brain tumor site and exert their tumoricidal effects.^14^ Currently, only 5% of clinically available drugs are capable of penetrating the BBB, underscoring the critical need for further investigation into both the BBB's mechanisms and potential therapeutic agents. In the context of medulloblastoma (MB), limited progress has been made with respect to enhancing survival outcomes through pharmacological interventions following chemotherapy.^15^ Notably, a pediatric study by Evans et al.^16^ demonstrated that vincristine treatment improved the survival rate of high‐risk MB patients aged 3–5 years from 65% to 79%. In another study, a regimen combining lomustine, cisplatin, and vincristine successfully inhibited tumor progression, leading to a remarkable 2‐year survival rate of 96% among children aged 3–10 years^17^, ^18^ However, although the use of these agents has improved survival rates, the long‐term survival remains limited due to the restrictive nature of the BBB. In addition, tumors affect the BBB integrity, resulting in a densely heterogeneous vasculature known as the blood‐tumor barrier (BTB). The BTB consists of both existing and newly formed blood vessels that supply nutrients and oxygen to the tumor, thereby facilitating the migration of cancer cells to other areas of the brain.^19^ Further factors, such as genetic and epigenetic instability^20^ and cellular heterogeneity^21^ contribute to the resistance of CNS tumors to current standard of care, that is, high‐dose radiotherapy (RT) or chemotherapy.^22^ Compounding these challenges, chemotherapeutic agents often suffer from limitations such as poor solubility and short half‐lives in the blood,^23^ while high‐dose RT may cause secondary risks to the patient's health.^24^ Given these significant barriers in brain cancer treatment—ranging from cancer localization and poor drug bioavailability to the protective nature of the BBB—innovative strategies are urgently needed to enhance treatment efficacy while minimizing systemic toxicity.

In this context, recent years saw nanomedicine make a substantial impact on the treatment of brain cancer, largely due to the unique chemical and physical properties of various metal nanoparticles (NPs),^25^ including gold nanoparticles (AuNPs). Gold, unlike other materials commonly employed in nanomedicine, emerged as a promising candidate for cancer therapy due to its inert nature, resistance to corrosion, and reactivity under physiological conditions. Notably, the unique capability to precisely control the shape and size of AuNPs during synthesis, along with the ease of surface chemistry modification, enhances their versatility and functionality.^26^ Equally important is the ability of AuNPs to undergo passive accumulation and preferential retention at tumor sites, a phenomenon driven by the enhanced permeability and retention (EPR) effect. This effect stems from the abnormal and leaky vasculature characteristic of tumor tissues, which facilitates NPs penetration, while impaired lymphatic drainage reduces clearance. Consequently, AuNPs can achieve higher concentrations within the tumor microenvironment, thereby enhancing their potential for targeted therapy and improving the efficacy of treatment strategies.^27^ Furthermore, AuNPs exhibit key properties such as biocompatibility, unique and tunable optical characteristics and the ability to cross the BBB.^28^, ^29^ One of the most important properties of AuNPs is their ability to interact with light through a phenomenon called localized surface plasmon resonance (LSPR). When these NPs are exposed to specific wavelengths of light, their electrons start to vibrate in sync, generating heat. This effect allows AuNPs to act as tiny, localized heat sources through plasmonic effects.^30^ This property is especially useful in cancer treatment, particularly in photothermal therapy (PTT). By adjusting the size and shape of the NPs, scientists can fine‐tune the light they absorb, ensuring they respond efficiently to near‐infrared (NIR) light. Since NIR light can penetrate deep into tissues, the heat generated by the NPs can be precisely focused on cancer cells, helping to destroy them while minimizing damage to surrounding healthy tissue.^31^ This is what happens in the work of Yu et al.,^32^ where AuNPs loaded with temozolomide (TMZ) (TMZ@GNPs) and functionalized with anti‐EphA3 were developed (anti‐EphA3‐TMZ@GNPs) for PTT (GNPs‐PPTT). This strategy aimed to address glioma resistance to TMZ and enhance the therapeutic efficacy for GBM. Following photothermal treatment, the survival time of nude mice with subcutaneous GBM in the anti‐EphA3‐TMZ@GNPs group was extended to 46 days, representing a 1.64‐fold increase compared to the TMZ‐only group. Joh et al.^33^ used as radiosensitizers in RT 12 nm‐diameter PEGylated AuNPs (PEG‐AuNPs) in both U251 glioma cells line and mouse brain endothelial cells compared to those without AuNPs. For in vitro radio sensitization experiments, GBM cells were exposed to 4 Gy irradiation (150 kVp), while for in vivo experiments, mice received 20 Gy (175 kVp). By targeted RT directed at tumor‐associated endothelial cells containing AuNPs, the tumor vasculature was unable to sustain nutrient supply to the tumor tissue, resulting in chemical necrosis. Furthermore, orthotopic U251 xenograft‐bearing mice treated with AuNPs followed by RT showed a median survival time of 28 days, compared to 14 days for mice that received RT alone.

In another study, Salamone et al.^34^ investigated thiol‐functionalized AuNPs loaded with methotrexate (MTX) as drug delivery probes, thanks to their small size and hydrophilic character. The nanoconjugate was tested in vitro on two neuroblastoma cells line SNJKP and IMR5, both characterized by high n‐Myc expression. Results showed that the AuNPs‐MTX nanoconjugate has a significantly greater effect than MTX alone, with permeation studies highlighting the role of AuNPs in facilitating deeper drug penetration into the cells.

Kim et al.^35^ synthesized nanoconjugates consisting of AuNPs linked to photosensitizers (PSs) through disulfide bonding between Chlorin e6 (Ce6) and glutathione‐coated AuNPs. To enable both oral absorption and targeted GBM delivery, PEGylated lactoferrin (Lf‐PEG) was added to the AuNPs surface, creating the Ce6‐AuNPs‐Lf complex. This study investigated how Ce6‐AuNPs‐Lf binds to Lactoferrin receptors (LfR) at the intestinal barrier and BBB, achieving efficient penetration through both. In an orthotopic GBM mouse model, Ce6‐AuNPs‐Lf demonstrated significantly enhanced oral bioavailability and GBM targeting, reaching 7.3 ± 1.2% and 11.8 ± 2.1 µg/kg, respectively. The sequence of applying photodynamic therapy followed by PTT was critical to optimizing treatment outcomes, as the AuNPs plasmonic properties increased reactive oxygen species (ROS) production. Consequently, oral administration of Ce6‐AuNPs‐Lf for GBM phototherapy achieved remarkable antitumor effects due to specific GBM targeting and improved photoconversion efficiency.

The promising characteristics of functionalized AuNPs position them as valuable tools in advancing brain tumor therapies. This review explores both conventional and innovative approaches for treating a range of brain cancers, including GBM, pediatric brain tumors, and brain metastases. Standard techniques such as RT, chemotherapy, and PTT are examined to provide context for how AuNPs might augment current treatment protocols. The review further delves into the intrinsic properties of metallic NPs that enhance their application in oncology. Particularly, their ability to cross the BBB represents a significant advancement, as the BBB is a critical regulatory and protective interface, controlling substance exchange between the bloodstream and brain tissue. By overcoming this barrier, NPs offer potential for targeted delivery of therapeutic agents directly to tumor sites, including drug bioavailability and reducing systemic side effects.

Ultimately, this review aims to highlight how AuNPs could transform brain tumor treatment by enhancing drug delivery, improving therapeutic efficacy, and enabling more precise targeting of tumor cells. While current studies demonstrated their potential in drug delivery, RT, and chemotherapy for GBM, future research is expected to focus on optimizing their design to further increase specificity and minimize off‐target effects. Ongoing clinical trials are also exploring the integration of AuNPs with advanced treatment modalities, such as immunotherapy and PTT, to harness synergistic effects and improve patient outcomes. Additionally, the development of AuNP‐based systems capable of crossing the BBB more efficiently and delivering multi‐drug combinations is a promising avenue. These advancements, alongside preclinical and clinical evaluations, are anticipated to provide robust evidence for their clinical translation, potentially offering safer, more targeted, and effective therapeutic options for brain cancer soon.

## 2 BRAIN CANCER AND STANDARD MEDICAL APPROACHES

Brain cancers consist of a diverse group of neoplasms originating from cells within the CNS, presenting as either benign or malignant.^36^ The most common primary intracranial tumor is GBM, comprising more than 50% of all gliomas,^37^ that develops from a small population of adult neural stem and progenitor cells.^38^ In parallel to GBM, solitary brain metastases are becoming increasingly common, especially with the increase in the elderly population all over the world.^39^ One major challenge is distinguishing GBM from metastatic tumors, a task that poses growing difficulties for neuroradiologists and neurosurgeons alike.^40^ Frequently, even with a documented history of cancer, a biopsy is essential to achieve histological confirmation.^41^ While most research and clinical attention have traditionally focused on adult brain tumors such as GBM and metastatic lesions, pediatric brain tumors pose equally critical challenges. They represent the leading cause of cancer‐related mortality and long‐term neurological deficits in children. Their diagnosis is often delayed due to the nonspecific nature of early symptoms, frequently resembling those of common pediatric conditions, and the limited ability of young patients to articulate their clinical experiences.^42^ Although less frequent than adult brain tumors, pediatric cases appear to follow distinct molecular trajectories, diverging from the classical model of tumorigenesis based on the progressive accumulation of unrepaired genetic mutations.^43^ Emerging evidence indicates that pediatric brain tumors are molecularly distinct from their adult counterparts, and our understanding of their tumor microenvironment remains limited.^44^

### 2.1 GBM

GBM is the most commonly occurring and aggressive malignant brain cancer in adults and accounts for 16% of all primary neoplasms of the CNS.^45^ GBM has always been associated with particularly high mortality rates: average survival rates vary from 5 to 15 months, and 5‐year survival rates as low as 5% in several studies.^46^, ^47^, ^48^

GBM most frequently manifests in the cerebral hemispheres' subcortical white matter, including the frontal (28.6%), temporal (25%), parietal (15.3%), and less commonly, occipital (3.9%) lobes.^49^

According to the World Health Organization (WHO),^50^ GBM is classified as grade IV tumor, characterized by extreme cellular abnormality, rapid proliferation, and frequently associated with necrosis of brain tissue, inducing microvascular proliferation within the affected region.^51^ Microvascular proliferation, a defining feature of GBM, arises as a response to the hypoxic microenvironment created by rapid tumor growth and necrosis. This process is driven by aberrant angiogenesis, primarily mediated by vascular endothelial growth factor (VEGF), which promotes the formation of structurally abnormal, hyperpermeable blood vessels. These disorganized vascular networks not only facilitate tumor progression by supplying oxygen and nutrients but also contribute to therapeutic resistance, making them a critical target for novel treatment strategies.^52^, ^53^

Initially, GBM was believed to originate solely from glial cells. However, recent evidence suggested that it may arise from a variety of cell types exhibiting neural stem cell‐like characteristics. These tumors are now classified into two categories: primary, which develop de novo, and secondary, which evolve from lower‐grade tumors over time.^54^

At biological level, gliomas often carry recurrent missense mutations in the isocitrate dehydrogenase enzymes IDH1 and IDH2.^55^ These enzymes play a key metabolic role by transforming isocitrate into α‐ketoglutarate (αKG) while simultaneously reducing NADP to NADPH. Specific IDH1 and IDH2 mutations are characteristic of distinct subtypes, including low‐grade gliomas, secondary GBMs, chondrosarcomas, intrahepatic cholangiocarcinomas, and certain hematologic cancers.^56^ Numerous preclinical models illustrated the tumorigenic effects of IDH1/2 mutations, which impact epigenetic regulation, tumor cell differentiation, and metabolic processes.^57^, ^58^ A rapidly advancing therapeutic strategy in precision oncology is the development of small‐molecule inhibitors specifically targeting mutant IDH1. This approach gained substantial clinical significance, exemplified by the recent regulatory approval of ivosidenib (AG‐120), the first selective mutant IDH1 inhibitor, for the treatment of IDH1‐mutated acute myeloid leukemia.^59^ These inhibitors function by blocking the aberrant enzymatic activity of mutant IDH1, which leads to the accumulation of the oncometabolite 2‐hydroxyglutarate (2‐HG), a driver of tumorigenesis. Expanding beyond hematologic malignancies, inhibitors targeting mutant IDH1 and IDH2 enzymes showed promise in preclinical studies and are currently undergoing clinical evaluation. This emerging drug class holds significant potential as a targeted therapeutic approach for gliomas, addressing a critical need for more effective treatments in these malignancies.^60^

The causes of GBM are not entirely clear yet. They could be high exposure to ionizing radiation first, environmental variables, smoking, nutritional or occupational risk factors, or even ovarian steroid hormones.^61^

As far as glioma diagnostics are concerned, computed tomography (CT), magnetic resonance imaging (MRI), and positron emission tomography (PET) are the most widely used techniques.^62^

**FIGURE 1:**
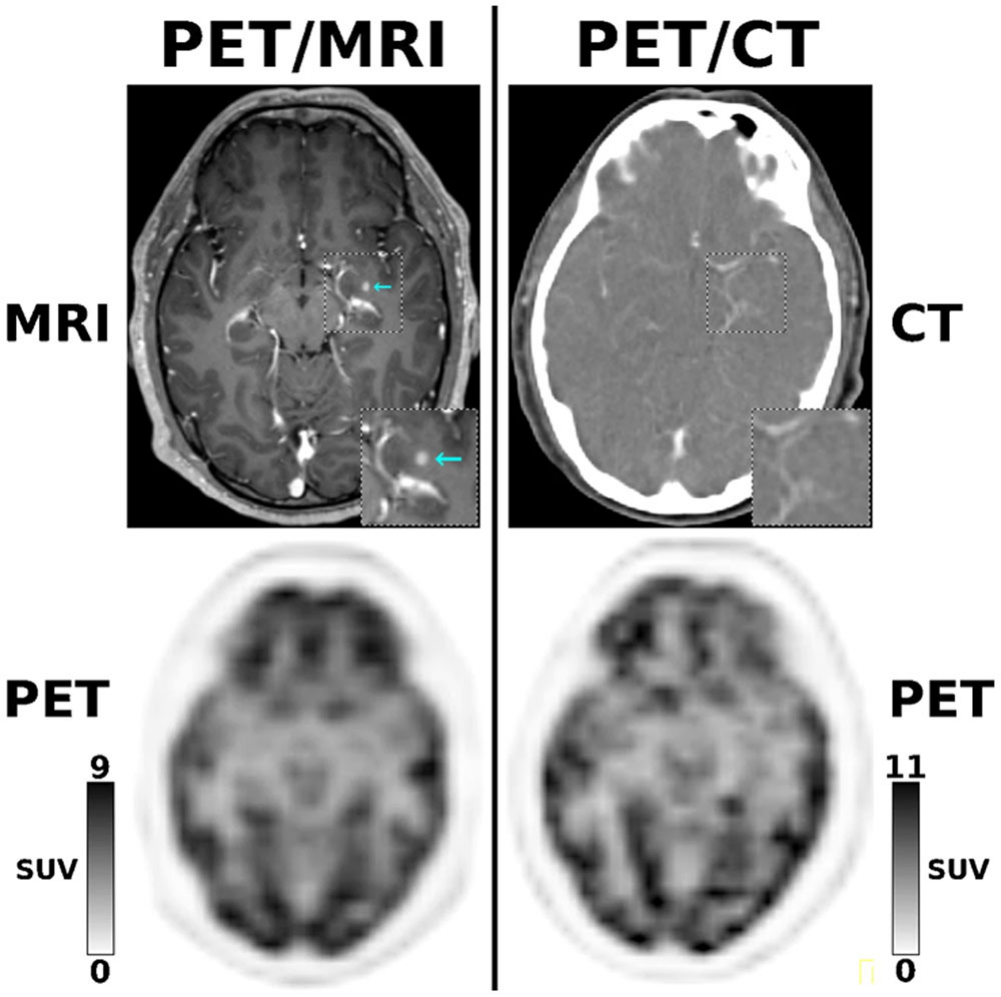
Imaging of a 61‐year‐old non‐small cell lung cancer (NSCLC) stage IV patient for staging before nivolumab. [18F]FDG‐PET is negative on both PET/MRI and PET/CT, but contrast‐enhanced MRI in PET/MRI identifies a small left hippocampal metastasis (cyan arrow), missed by contrast‐enhanced CT in PET/CT, warranting RT.^65^ Abbreviations: CT, computed tomography; MRI, magnetic resonance imaging; PET, positron emission tomography. [Color figure can be viewed at wileyonlinelibrary.com]

CT, typically performed at initial presentation, may reveal a poorly defined hypodense lesion with occasional hyperdense regions reflecting increased cellular density or intratumoral hemorrhage. However, the diagnostic specificity of standard CT is limited, though cerebral perfusion mapping can enhance its utility in evaluating tumor vascularity.^63^ MRI, in contrast, offers superior spatial resolution and tissue characterization, making it an indispensable tool for tumor staging, grading, and treatment planning. Both contrast‐enhanced and non‐contrast MRI provide detailed structural and functional information, with advanced techniques such as diffusion‐weighted and perfusion‐weighted imaging further elucidating microstructural and hemodynamic features of the tumor.^64^ When combined with MRI, PET imaging adds valuable metabolic data. For example, Figure 1 illustrates this diagnostic advantage, showing a 61‐year‐old patient with stage IV non‐small cell lung cancer. While 18F‐fluorodeoxyglucose ([18F]FDG)‐PET findings were unremarkable on both PET/MRI and PET/CT, the contrast‐enhanced MRI portion of PET/MRI detected a small brain metastasis in the left hippocampus, which was not visible on the contrast‐enhanced CT portion of PET/CT. This finding underscored the need for targeted radiation therapy.^65^

The use of 18F‐fluoroethyltyrosine (FET) PET, in particular, demonstrated high specificity in distinguishing tumor progression from treatment‐related changes like pseudo progression or necrosis. The integration of radio genomic analyses and machine learning algorithms into imaging protocols further enhances the precision of GBM characterization, allowing for more personalized diagnostic and therapeutic strategies.^66^

In this diagnostic landscape, a specimen of the tumor mass by biopsy is often required to have a better diagnostic glioma characterization. In this context, liquid biopsy gained attention as a promising, noninvasive diagnostic approach that complements traditional methods. By detecting and quantifying tumor‐derived components, such as circulating tumor DNA (ctDNA), RNA, proteins, or extracellular vesicles, in biofluids like blood or cerebrospinal fluid, liquid biopsy provides valuable insights into tumor biology. This technique not only aids in initial diagnosis but also offers potential for monitoring disease progression, therapeutic response, and the emergence of resistance, thereby enhancing the precision and scope of GBM management.^67^ In comparison, standard brain biopsy is considered an invasive procedure with notable limitations. Risks such as infection, bleeding, and neurological complications are inherent to the approach, and the possibility of sampling bias further undermines its comprehensiveness.^68^

**FIGURE 2:**
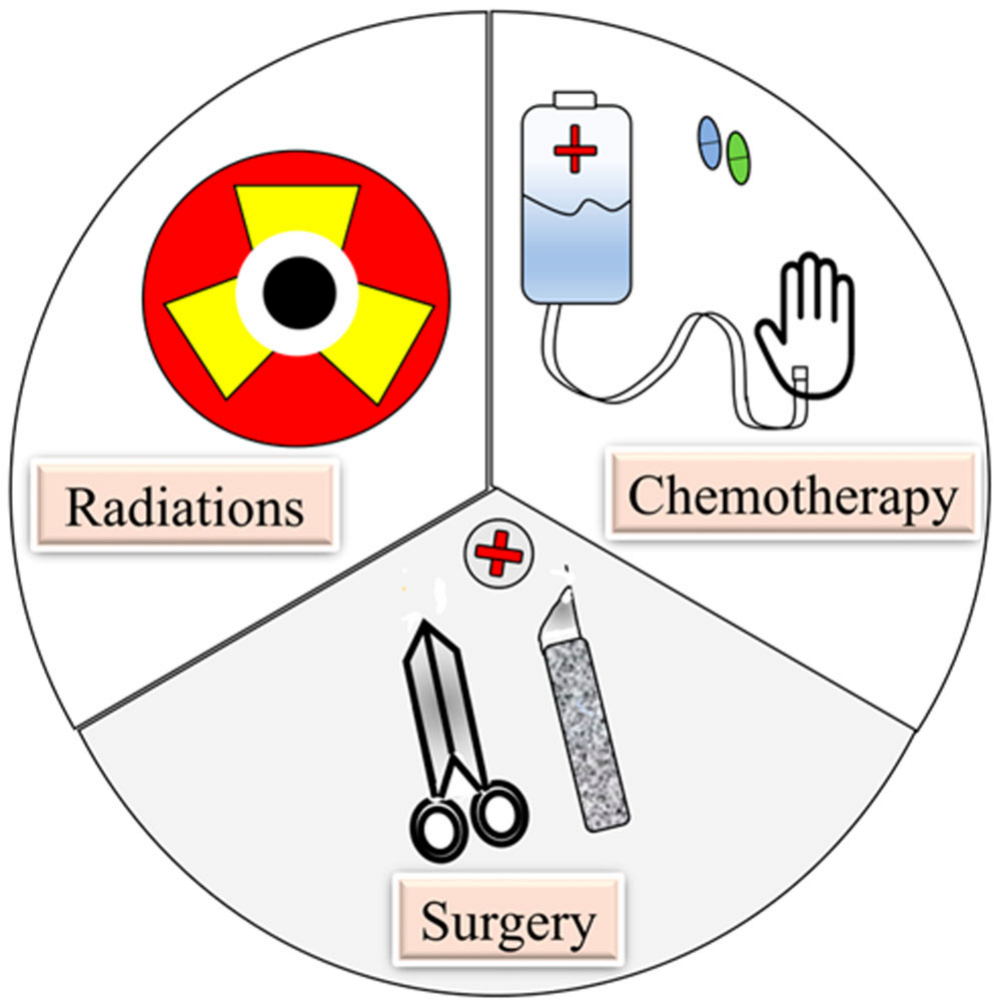
Standard medical approaches for GBM treatment: RT, chemotherapy and surgical removal.^61^ [Color figure can be viewed at wileyonlinelibrary.com]

The main vehicle used to attempt a possible cure from GBM is surgical resection.^69^ Unfortunately, the margins of the tumor are often not well defined, or the host site of the mass does not support the operation. To address the challenge of poorly defined tumor margins, an oncological fluorescence technique was introduced as an intraoperative diagnostic tool. This approach aims to enhance the precision and effectiveness of surgical interventions by improving tumor delineation during surgery.^70^ Precise differentiation between neoplastic tissue and normal brain parenchyma remains particularly challenging at the invasive tumor margins, where a significant number of tumor cells infiltrate beyond the radiologically enhanced region, often in areas where the BBB is still intact. In response, fluorescence‐guided surgery gained traction in glioma resection. Two primary agents were employed for this purpose: 5‐aminolevulinic acid (5‐ALA), a metabolic fluorophore, and sodium fluorescein (SF), which selectively accumulates in tumor tissue due to BBB disruption. Notably, SF can extend beyond gadolinium‐enhanced areas, likely due to its smaller molecular size, offering improved visualization of infiltrative tumor regions.^71^ Following surgical resection, where feasible, patients typically undergo RT, chemotherapy, or a combined radio‐chemotherapy regimen^72^ (Figure 2).

Chemotherapy often includes TMZ, a radiomimetic agent recognized as the primary treatment for brain tumors,^73^ including GBM, since 2005. TMZ, commercially known as Temodar or Temodal, is a lipophilic prodrug belonging to the imidazotetrazine class. Its ability to cross the BBB makes it suitable for oral administration, a significant advantage in treating brain malignancies. TMZ remains stable in acidic environments but undergoes activation at physiological pH, where it is converted into its active metabolite, 5‐(3‐methyltriazen‐1‐yl) imidazole‐4‐carboxamide (MTIC). MTIC is further hydrolyzed to generate methyldiazonium ions, highly reactive electrophilic molecules that induce DNA damage by methylating guanine residues, ultimately disrupting tumor cell replication and survival.^74^ However, the extensive use of TMZ, combined with the highly heterogeneous and mutation‐prone nature of GBM, frequently leads to the development of resistance against this chemotherapeutic agent. Alarmingly, more than half of GBM patients fail to respond to TMZ treatment, and predictive biomarkers for therapy effectiveness remain limited, with O6‐methylguanine‐DNA methyltransferase (MGMT) promoter methylation being one of the few established indicators. MGMT is a DNA repair enzyme that counteracts the cytotoxic effects of TMZ by removing alkyl adducts from the O6 position of guanine, thereby preventing DNA damage and apoptosis.^75^ When the MGMT promoter is methylated, gene expression is silenced, reducing the enzyme's activity and increasing the tumor's sensitivity to TMZ. Conversely, in tumors with an unmethylated MGMT promoter, MGMT is actively expressed, leading to efficient DNA repair and subsequent resistance to TMZ.^76^

The challenge of addressing TMZ resistance is further compounded by its dual nature: it may be an intrinsic property of certain tumors with high MGMT expression or emerge as an adaptive mechanism following initial therapy.^77^

Moreover, the cellular and molecular heterogeneity observed in GBM is largely attributed to specific subpopulations of tumor stem cells (TSCs), also known as tumor‐initiating cells, which play a central role in treatment resistance, showing marked resilience against both TMZ and RT.^78^ In recent years, significant advancements were made in the identification and characterization of TSCs, paving the way for the development of innovative diagnostic and therapeutic strategies for brain tumors. Notable research uncovered critical aspects of brain TSCs, including their heterogeneity and unique immunobiological properties, offering new avenues for targeted therapeutic interventions and improved clinical outcomes.^79^ The main approach used to target the TSCs is the use of NPs‐encapsulated drugs functionalized with antibodies/ligands targeting simultaneously two or more TSCs markers. Recent advancements in NPs‐based strategies introduced innovative approaches to overcome the challenges posed by GBM stem cells. Multifunctional NPs were developed to deliver chemotherapeutics, gene therapies, or immunomodulators with enhanced specificity and efficiency. One promising direction involves NPs functionalized with ligands targeting markers such as CD133, SRY‐box transcription factor 2 (SOX2), and integrin α6, enabling precise recognition of GBM stem cells. Additionally, NPs designed to release their payload in response to the tumor microenvironment, such as acidic pH or enzymatic activity (e.g., metalloproteinases), showed significant potential in preclinical models.^80^, ^81^

In Table 1, it exhibited a summary comparison of standard medical approaches to cancer diagnosis and treatment.

**TABLE 1:** Comparative analysis of standard medical approaches for cancer diagnosis and treatment.

Category	Strengths	Weakness	Common use
Treatment: RT	Effective for local tumor control	Side effects such as fatigue, cognitive decline	Standard in GBM treatments protocols
Treatment: Chemotherapy	Standard care, effective when combined with RT	Systemic side effects, limited efficacy as monotherapy	Used with RT in newly diagnosed GBM
Treatment: Immunotherapy	Harnesses immune system to target tumor	Limited efficacy, potential for severe immune reactions	Emerging treatment, clinical trials
Treatment: Experimental Therapies	Innovative, potential for targeted and gene therapies	Experimental, not widely available, or proven yet	Under investigation for personalized medicine
Diagnostic: MRI	High‐resolution imaging, detailed structural info	High cost, contraindicated in patients with metal implants	Tumor localization, surgical planning
Diagnostic: CT scan	Quick, widely available, good for emergencies	Radiation exposure, less detailed than MRI	Emergency situations, basic tumor identification
Diagnostic: PET scan	Functional imaging, metabolic activity assessment	Limited availability, high cost, and radiation exposure	Research and detailed metabolic activity evaluation
Diagnostic: Biopsy	Definitive diagnosis, direct tissue analysis	Invasive, potential risks like bleeding or infection	Confirmatory diagnosis, molecular profiling
Diagnostic: Liquid Biopsy	Minimally invasive, potential for early detection	Limited sensitivity	Research and experimental early diagnosis

Abbreviations: CT, computed tomography; GBM, glioblastoma; MRI, magnetic resonance imaging; PET, positron emission tomography.

### 2.2 Brain metastases

Brain metastases are frequently observed in patients with progressed solid tumors.^82^, ^83^, ^84^

As primary tumors grow, genetic and genomic instability leads to the emergence of cells that adopt unusual traits, eventually forming distinct metastatic subpopulations with largely irreversible characteristics. These cells proceed sequentially through a series of steps following their initial invasion, including entry into the bloodstream (intravasation), survival as circulating tumor cells (CTCs) within bodily fluids, arrest at distant sites, exiting the bloodstream (extravasation), formation of micro‐metastases, and, ultimately, colonization of secondary organs.^85^, ^86^

CNS metastases are categorized into parenchymal, leptomeningeal, and epidural types, the latter involving the spinal region. Each of these specialized microenvironments presents unique conditions that CTCs may encounter as they travel through the bloodstream, cerebral lymphatics, or cerebrospinal fluid. When solitary CTCs cross the BBB, they face the complex transition from early micro metastases formation to the establishment of larger, stable macro metastases, requiring adaptation to the unique CNS environment.^87^, ^88^

A crucial process regulating invasion and metastasis is the epithelial‐mesenchymal transition (EMT).^89^ During EMT, cells lose their polarity and cell‐cell adhesion, and undergo changes of cell surface and cytoskeletal proteins, enhancing their migratory and invasive abilities.^90^, ^91^, ^92^

Nowadays, as standard approaches for the treatment of metastatic brain cells are the stereotactic irradiation (STI), because it allows high doses to the lesion from multiple directions while minimizing the delivery to surrounding healthy tissue,^93^ and whole brain RT (WBRT), that is however very aggressive and harmful in patients with poor expected survival.^94^ Moreover, the combination of WBRT and surgery targets microscopic disease at the original tumor site, effectively reducing the risk of brain recurrences, though it does not increase overall survival in all cases.^95^ In detail, for patients with a single brain metastasis, combining WBRT with surgical intervention has been associated with better survival rates than WBRT alone; conversely, in patients with multiple brain metastases, the effectiveness of surgical removal appears limited and often contradictory.^96^

### 2.3 Pediatric brain cancer

Pediatric brain tumors are the leading cause of death in children worldwide.^97^, ^98^ The 10‐year survival rate for children with brain cancer between 0 and 19 years is approximately 72%, with GBM having the lowest survival rate at 17%, and pilocytic astrocytoma showing the highest at 96%. Notably, tumors in the brainstem have the poorest survival outcomes, while those affecting cranial nerves demonstrate the best prognosis.^99^ Additionally, survival rates improve with the child's age at diagnosis, as younger patients often cannot endure the same level of treatment intensity as older children.^100^

Malignant gliomas, meningiomas, pituitary tumors, and metastases represent the most frequent brain tumors in adults,^101^ though they are rare among pediatric cases.^102^, ^103^ Conversely, benign gliomas, primary neuroectodermal tumors, and craniopharyngiomas make up a larger proportion of brain tumors observed in young children compared to the adult population.^104^

Additionally, notable differences exist in the anatomical distribution of brain tumors between age groups: in adults, most brain tumors develop within or near the cerebral hemispheres, while in children older than 1 year, approximately 50% of tumors are located infratentorially.^105^

In 2016, the WHO updated its classification system to include the amplification status of the chromosome 19 microRNA cluster (C19MC) region on chromosome 19 as a criterion,^106^ categorizing all embryonal CNS tumors as malignant and of grade IV.^104^

For pediatric tumor treatment, surgery plays a key role in improving survival rates for high‐grade gliomas, though some cancer cells may remain because of the tumor's invasive spread. However, when patients are not candidates for complete resection due to the infiltrative nature of the tumor or because of its location,^107^ RT is often used to further extend survival^108^ by easing symptoms, which can greatly enhance the child's quality of life, depending on cancer type.^109^ In particular, the use of a volume‐targeted technique using image‐guided radiation therapy was included for more effective targeting of the tumor area.^110^

Chemotherapy with TMZ is less frequently recommended, as studies have shown only limited improvements in survival outcomes for children.^111^ In fact, numerous chemotherapeutic agents can cause adverse effects on the CNS, particularly when administered intrathecally or at high doses, with common side effects including encephalopathy, cerebellar degeneration, myelopathy, and alterations in sensory perceptions such as vision, hearing, and taste.^112^, ^113^

## 3 PHYSIO‐CHEMICAL PROPERTIES OF AUNPS

The term “nanomaterial” typically refers to materials with dimensions less or equal to 100 nm.^114^, ^115^ Specifically, when particles attain the nanoscale, with sizes comparable to the de Broglie wavelength of charge carriers or the wavelength of light, the loss of crystalline periodicity and alterations in surface atomic density result in marked differences in their properties relative to bulk materials.^116^ This is mainly due to three factors: (a) nanomaterials exhibit high surface to volume ratio and a greater particle density per unit mass, (b) the proportion of atoms located at the surface is higher, and (c) surface atoms in nanomaterials have fewer neighboring atoms.^117^

Furthermore, over the last decade, significant focus was placed on noble metal NPs due to their ability to support LSPR phenomenon. LSPR involves the oscillation of free electrons (referred to as plasmons) in the conduction band, which occurs when metallic nanostructures interact with incident light. The collective movement of these electrons leads to amplified optical absorption and scattering in structures smaller than the wavelength of the incoming light, with their spectral characteristics being influenced by factors such as the material, size, shape, charge distribution, and surrounding environment.^118^ Additionally, the LSPR effects observed in noble metal NPs are particularly well‐documented, as their resonance frequencies lie within both the visible and near‐infrared regions, making them a focus of research for the development of biomedical technologies.^119^

Given the reliance of the LSPR effect on the properties of NPs, tuning their characteristics is essential for optimizing the performance of plasmonic NPs. The current approaches for fine‐tuning the LSPR properties of metallic NPs typically involve modifying the particle morphology, such as altering their size, shape, aspect ratio (i.e., nanorods or nanoplates), and branching (i.e., nanostars).^120^

**FIGURE 3:**
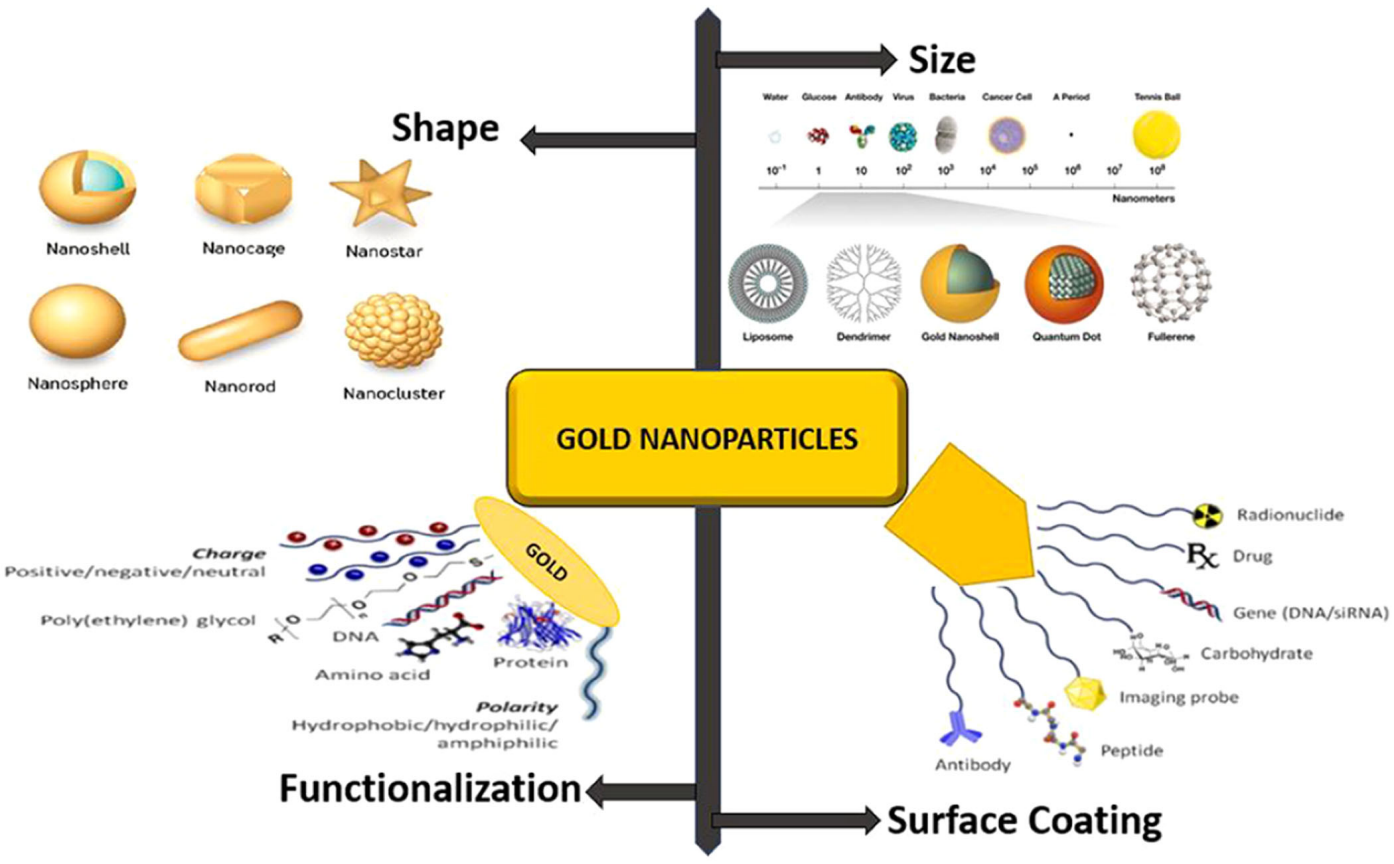
The unique physical and chemical properties of AuNPs. AuNPs own such properties: morphological variation from spherical to multispikes structure; functionalization with antibodies, carbohydrates, DNA/RNA, and peptides; drug loading for medical applications.^122^ [Color figure can be viewed at wileyonlinelibrary.com]

Among the noble metals, gold (Au) is certainly one of the most important owing to its unique physical and chemical properties^121^ (Figure 3).

Firstly, due to its high atomic number (*Z* = 79), AuNPs exhibits elevated photoelectric absorption coefficients,^123^ thereby enhancing its antitumor efficacy in RT.^124^ This increased absorption of X‐rays leads to a larger cross‐section for cancerous cells, which helps to protect surrounding healthy tissues. Additionally, the interaction of gold with X‐rays results in the emission of photoelectrons, Compton electrons, and other secondary electrons,^125^ which have the ability to directly ionize DNA within cells, so let AuNPs act as “radiosensitizers.”^126^

Another of the most AuNPs significant property being their ability to form composites with various materials,^127^ such as organic compounds or inorganic carbon. This capability not only allows for precise tuning of their structural and functional characteristics but also enables better control over their cost‐effectiveness and performance. Furthermore, when combined with other metals, including metallic oxides or hydroxides, AuNPs at low concentrations shown reduced toxicity to normal cells while demonstrating enhanced antimicrobial activity, particularly against bacteria.

In Hu et al. study,^128^ ultrasmall AuNPs (UsAuNPs) were synthesized on ultrathin 2D metal‐organic frameworks (MOFs) through in situ reduction, resulting in the creation of a hybrid material (UsAuNPs/MOFs). This hybrid structure combined the unique properties of UsAuNPs with the advantages of the ultrathin 2D MOFs, exhibiting remarkable peroxidase‐like activity, particularly in the decomposition of hydrogen peroxide (H_2_O_2_) into highly reactive hydroxyl radicals (·OH). The UsAuNPs/MOFs nanozyme demonstrated excellent antibacterial efficacy against both Gram‐negative *Escherichia coli* and Gram‐positive *Staphylococcus aureus* bacteria, even at low concentrations of H_2_O_2_. These results highlighted the promising potential of this hybrid nanozyme as an effective antibacterial agent, suggesting its future application in antibacterial therapy and clinical settings.

All the AuNPs unique properties led to extensive studies of Au composites as promising agents for antimicrobial applications.^129^, ^130^ The ability to alter the surface biochemistry of NPs holds significant potential for enhancing the cellular uptake of specific compounds or pharmaceuticals. Moreover, by conjugating AuNPs with therapeutic agents, their effectiveness can be improved, as such modifications enable targeted delivery of the NPs‐drug complex to tumor cells.^31^

Nosrati et al.^131^ experimented AuNPs and drug‐loaded, mPEG‐conjugated curcumin (mPEG‐CUR) self‐assembled NPs (mPEG‐CUR@Au) and evaluated their potential as drug carriers and radiosensitizers in an orthotopic breast cancer mouse model. The 4T1 mammary carcinoma cells were injected into the mice, and the mPEG‐CUR@Au NPs were administered intravenously once the tumors reached the desired size. The results demonstrated a significant enhancement in cancer treatment efficacy through the use of these all‐in‐one NPs, which facilitated synchronous chemoradiotherapy. This was confirmed by evaluating cell viability, cell proliferation, and ROS production. In vivo anticancer experiments further revealed that the mPEG‐CUR@Au NPs significantly increased the radiation sensitivity of 4T1 mammary carcinoma cells, contributing to the effective suppression of breast cancer growth.

Koley et al.^132^ developed stable p‐sulfonatocalix[6]arene‐functionalized AuNPs (SCx6AuNPs) with an approximate size of 7.5 nm and investigated their potential for efficient drug delivery and controlled release of doxorubicin (Dox), an anticancer agent. These Dox‐loaded SCx6AuNPs demonstrated notable responsiveness to various external stimuli, including pH changes, spermidine, and adamantylamine, which acted as competitive binding agents to trigger drug release. To assess the cytotoxic effects of the synthesized SCx6AuNPs, free Dox, Dox‐loaded SCx6AuNPs, and other multicomponent systems, an MTT assay was performed using both the human diploid fibroblast WI26 cell line and the human lung carcinoma A549 cell line. The results indicated that the Dox‐loaded SCx6AuNPs exhibited reduced cytotoxicity, particularly toward normal cells, while maintaining effective drug release under multiple stimuli. These findings suggested that SCx6AuNPs are promising candidates for selective drug delivery, offering the potential for targeted and controlled treatment strategies.

**FIGURE 4:**
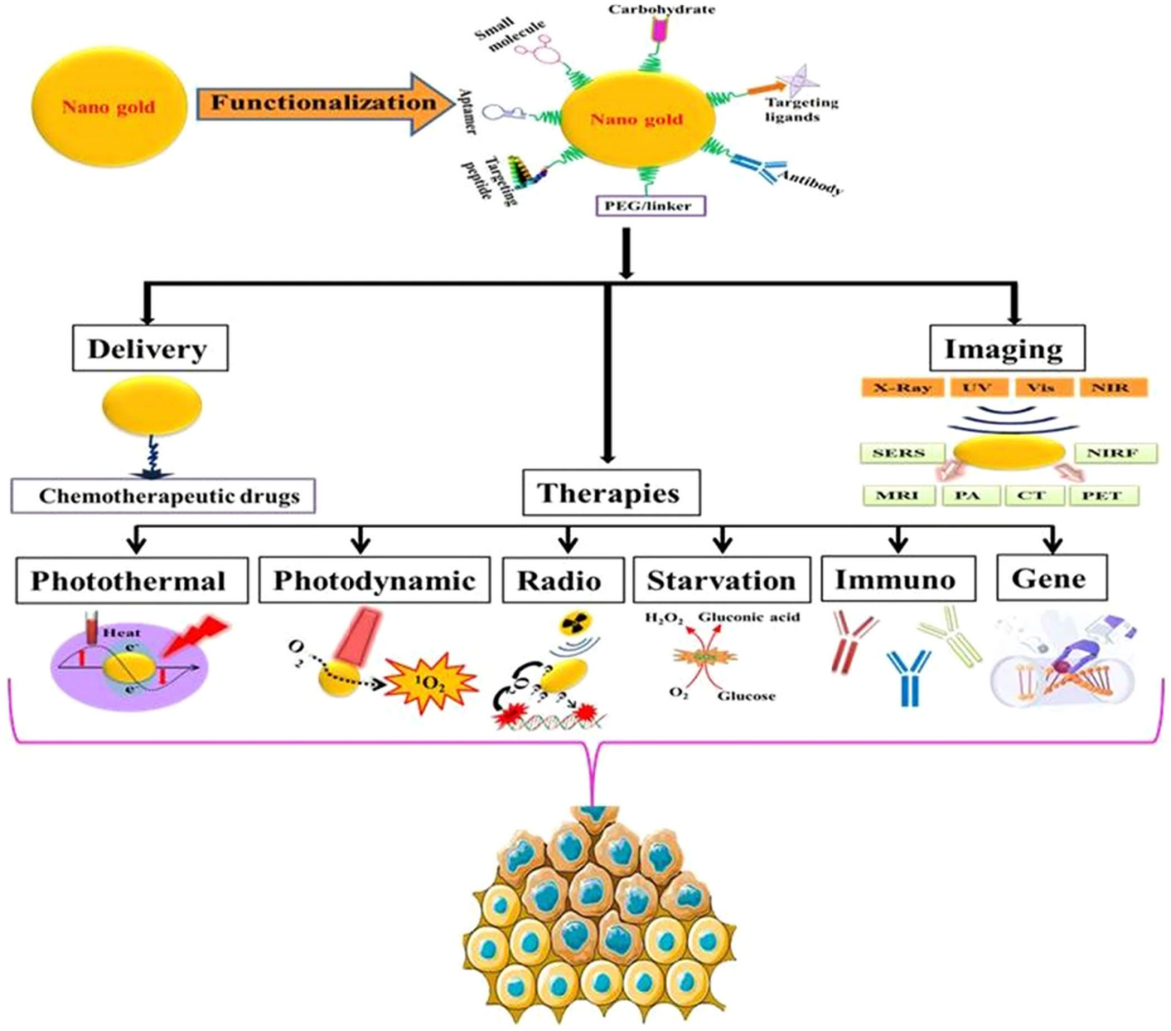
Medical applications of AuNPs in cancer theragnostic: imaging, therapies and drug delivery.^135^ [Color figure can be viewed at wileyonlinelibrary.com]

Finally, nanomaterials used in medical applications, such as for injections, must exhibit both chemical stability and biocompatibility,^133^ by meaning that the interaction between the nanomaterial and the host does not result in negative effects, including oxidative damage, genetic alterations, mutations, or programmed cell apoptosis.^134^ However, their potential toxicity remains a subject of ongoing debate, primarily because nanomaterials interact with mammalian cells and tissues in ways that differ from bulk materials. Metal‐based nanomaterials, in particular, can pose a risk due to their ability to release metal cations, which can damage cell membranes. On the other hand, due to their high reduction potential, AuNPs exhibit inertness and biocompatibility, reducing the likelihood of ionization and toxicity (Figure 4).^136^

In the work of Kadhim et al.,^137^ they aimed to assess the toxicity of AuNPs through both in vitro and in vivo approaches. The cytotoxicity of AuNPs was evaluated using an MTT assay on the rate embryonic fibroblast (REF) cell line; in parallel, an in vivo model was employed where AuNPs were administered intraperitoneally to male mice weighting between 25 and 30 g at a concentration of 100 µg/kg. The results of the in vitro cytotoxicity assay revealed no significant toxic effects or morphological alterations in the REF cells treated with AuNPs at concentrations of 1, 5, and 10 µg/mL. Similarly, the histopathological examination of tissues from the in vivo study showed no noticeable changes, indicating that AuNPs did not induce detectable toxicity at the tested dose.

Sulaiman et al.^138^ investigated the potential of hesperidin, a flavonoid glycoside known for its anticancer properties but limited by poor solubility, by loading it onto AuNPs (Hsp‐AuNPs). The cytotoxic effects of the Hsp‐AuNPs were assessed on the human breast cancer cell line MDA‐MB‐231 using MTT and crystal violet assays. The findings demonstrated a substantial reduction in cell proliferation and growth inhibition in the treated cancer cells, with these effects being more pronounced when compared to the normal human breast epithelial cell line (HBL‐100). This study suggested that Hsp‐AuNPs could enhance the therapeutic potential of hesperidin for cancer treatment.

## 4 NANOMEDICINE IN BRAIN ACTIVITY

Nanotechnology dramatically reshaped the study and treatment of brain function, offering groundbreaking advances in both diagnostics and therapeutics. This rapidly emerging interdisciplinary field, known as nano neuroscience, utilizes the unique properties of nanomaterials, such as their small size, biocompatibility, and ability to cross the BBB, to create novel solutions for treating CNS disorders. By enhancing drug delivery systems, nanotechnology holds particular promise for treating complex neurological diseases like Alzheimer's, Parkinson's, and brain cancer, where traditional therapies face major obstacles in penetrating the BBB and effectively targeting the brain.^139^

In the realm of brain cancer, particularly GBM, nanotechnology proved to be a transformative tool. GBM presents significant challenges due to its rapid growth and the protective nature of the BBB, which prevents most conventional drugs from reaching the tumor site. However, NPs, with their ability to be precisely engineered for size, surface charge, and targeting capabilities, can bypass the BBB and deliver therapeutic agents directly to tumor cells. This targeted approach not only enhances drug efficacy but also reduces systemic toxicity and side effects, offering a more effective treatment option for brain cancer patients.^140^, ^141^ Through such innovations, nanotechnology is paving the way for more personalized, efficient, and safer therapies in the fight against brain tumors.

NPs used in brain cancer treatment can be functionalized with specific ligands or antibodies that target tumor‐specific receptors, such as epidermal growth factor receptors (EGFR), which are commonly overexpressed in GBM. This allows for highly targeted drug delivery, improving treatment outcomes while sparing healthy brain tissue. AuNPs, for example, were widely studied for their ability to deliver chemotherapy drugs directly to brain tumor cells, enhancing their accumulation at the tumor site and reducing the adverse effects typically seen with conventional chemotherapy.^142^, ^143^, ^144^

In addition to drug delivery, nanotechnology also plays a critical role in the early detection and imaging of brain tumors. NPs can be used as contrast agents in imaging techniques like MRI, PET, and CT. These agents improve the resolution and sensitivity of imaging, making it possible to detect tumors at earlier stages and monitor their progression more accurately.^145^

Beyond drug delivery and imaging, nanotechnology also offers promising therapeutic strategies for brain cancer. AuNPs, when exposed to light, can convert light energy into heat, a property that can be harnessed in PTT to selectively destroy tumor cells while minimizing damage to surrounding healthy tissue. Additionally, gene therapies utilizing NPs to deliver small interfering RNA (siRNA) or microRNA showed potential in silencing genes that promote tumor growth, further inhibiting the progression of brain cancer. These innovative approaches represent a new frontier in the treatment of brain tumors, providing hope for more effective and personalized therapies.^146^ Nanotechnology holds significant promise for improving brain cancer diagnosis and treatment, but challenges remain in translating it to clinical practice. Issues such as tumor heterogeneity, brain tissue complexity, and NPs toxicity need to be addressed. Nevertheless, advancements in NPs design and a better understanding of brain interactions could enhance outcomes. The convergence of neuroscience and nanotechnology could revolutionize brain disorder treatments, leading to more personalized therapies.

### 4.1 BBB penetration

The BBB is a crucial and highly selective interface that separates the systemic circulation from the CNS, acting as a protective shield for the brain. This barrier is vital for maintaining the brain's delicate homeostasis, ensuring that harmful substances, such as toxins, pathogens, and fluctuations in ion concentrations, are prevented from entering the CNS. The BBB comprises specialized endothelial cells that form the inner lining of brain micro vessels, connected by tight junctions that significantly limit paracellular transport, thus restricting the movement of most substances between the blood and the brain. The endothelial cells are supported by a basement membrane and pericytes, which contribute to the structural stability of the barrier. Astrocytic end‐feet surround the capillaries, providing biochemical support and helping to maintain the integrity of the barrier. This highly organized structure forms a selective permeability barrier that allows essential nutrients like glucose and amino acids to pass through while blocking larger molecules, harmful chemicals, and pathogens from entering.^147^

While the BBB is essential for protecting the brain, it also represents a major challenge in the field of drug delivery. The rigid selectivity of the BBB prevents most therapeutic agents, particularly large molecules and NPs, from crossing into the CNS.

The BBB selectively permits the passage of lipophilic compounds with a molecular weight below 400–500 Da, effectively blocking nearly 98% of small molecules and larger therapeutic agents, such as monoclonal antibodies, antisense oligonucleotides, and viral vectors.^148^ For instance, TMZ, a key drug for GBM treatment, meets the molecular weight and lipophilicity criteria (194 Da), yet it achieves a tumor‐to‐blood concentration ratio of less than 20%. Additionally, active efflux mechanisms, such as P‐glycoprotein (P‐gp) and breast cancer resistance protein, further diminish the drug's efficacy by pumping it back into systemic circulation.^149^, ^150^

To address these challenges, researchers explored various strategies to enhance drug penetration across the BBB. One approach is passive diffusion, which allows lipophilic molecules with a molecular weight below 500 Da and an appropriate partition coefficient (logP between 0.5 and 6.0) to passively diffuse through endothelial cell membranes. However, larger or hydrophilic molecules typically cannot cross the BBB through this mechanism.^151^

Active transport mechanisms are also crucial for improving drug delivery. Receptor‐mediated transcytosis and adsorptive‐mediated endocytosis are two key strategies that utilize the BBB's natural transport systems. Receptor‐mediated transcytosis involves the binding of molecules to specific receptors on the endothelial surface, leading to their internalization and targeted transport across the barrier, which is particularly useful for delivering receptor‐specific drug‐ligand conjugates.^152^ Adsorptive‐mediated endocytosis relies on electrostatic interactions between positively charged NPs and the negatively charged endothelial membranes, facilitating their uptake and passage through the BBB.^153^

NPs, particularly AuNPs, garnered significant attention due to their ability to exploit these transport mechanisms. Their size, shape, surface charge, and surface functionalization make them highly adaptable for BBB penetration. AuNPs smaller than 20 nm are more efficient at crossing the BBB, as they are small enough to utilize vesicular transport pathways like receptor‐mediated transcytosis.^154^ Among the key receptors facilitating this process are transferrin receptors, which mediate iron transport and are commonly exploited for NPs delivery, low‐density lipoprotein receptors, involved in lipid metabolism, and insulin receptors (IR), which regulate glucose homeostasis and have been used for enhancing NPs uptake into the brain.^155^

The surface charge of AuNPs is another critical factor: neutral or slightly positively charged particles tend to show higher permeability through the BBB compared to highly positively charged ones, which may cause cytotoxicity or immune activation. On the other hand, negatively charged particles are often repelled by the endothelial surface. By functionalizing AuNPs with specific targeting ligands, their uptake can be significantly enhanced through receptor‐mediated mechanisms.^156^

Moreover, surface coatings like polyethylene glycol (PEG) can be used to extend the circulation time of AuNPs by preventing their rapid clearance from the bloodstream, thereby improving the chances of their reaching the BBB and being transported across it.^157^ PEGylation has been shown to significantly prolong systemic circulation by reducing opsonization and uptake by the reticuloendothelial system (RES), thereby increasing NPs accumulation at the target site. The molecular weight and density of PEG chains play a crucial role in determining the pharmacokinetics of AuNPs, influencing both their half‐life and biodistribution.^158^

Furthermore, functionalization with hyaluronic acid (HA) enhances BBB penetration through receptor‐mediated interactions, particularly targeting CD44 receptors, which are overexpressed in brain endothelial cells. This functionalization also improves biocompatibility and reduces systemic toxicity, making HA‐coated AuNPs a promising candidate for targeted drug delivery to the brain.^159^ The shape of the NPs also plays a significant role in their ability to cross the BBB. Rod‐shaped or elongated AuNPs, for example, have shown improved endocytosis and transcytosis compared to spherical NPs, likely due to more favorable interactions with the cell membrane and enhanced uptake mechanisms.^160^

Thus, the ability of AuNPs to penetrate the BBB depends on a combination of their physicochemical properties and the exploitation of specific biological mechanisms. By optimizing these properties, AuNPs provide a promising platform for drug delivery to the brain, potentially offering new therapeutic options for treating neurological disorders, including neurodegenerative diseases. However, further research into the long‐term safety and efficacy of these NPs is essential to harness their potential fully in clinical applications.

### 4.2 Active targeting in brain cancer therapy

Chemotherapy remains a cornerstone in the treatment of brain cancer, but the BBB often obstructs therapeutic drugs from reaching the brain, leading to limited treatment success. AuNPs emerged as a promising solution, as they can be engineered to carry various therapeutic molecules. This approach improves the solubility and stability of drugs while reducing their side effects. AuNPs offer several advantages, including biocompatibility, low immunogenicity,^161^ ease of synthesis, and unique optical properties such as surface plasmon resonance. These properties make them highly suitable for therapeutic applications, including PTT. Glioma, a highly aggressive and prevalent intracranial tumor, showed increasing mortality rates in recent years. To address the challenges posed by its complex microenvironment, AuNPs are widely utilized to optimize drug delivery to tumor sites. Recently, Sahli et al. demonstrated that hybrid AuNPs could be loaded with TMZ, gemcitabine, and decitabine using electrostatic interactions. These NPs effectively transported the drugs to U87 malignant glial cells, overcoming glioma cell resistance to TMZ and enhancing therapeutic outcomes through the synergistic action of the three drugs.^162^ Ruan et al. demonstrated that DOX could be conjugated to AuNPs using an acid‐sensitive hydrazone linker, with additional functionalization by angiopep‐2 to enhance BBB penetration. Both in vitro and in vivo studies showed that this gold‐based nanosystem improved drug delivery efficiency compared to free DOX.^163^ Subsequently, the same authors advanced the system by developing a new approach where small AuNPs were grafted onto gelatin NPs and modified with DOX, Cy5.5, and a dual peptide system combining Arg‐Gly‐Asp (RGD) and octarginine to further enhance BBB crossing. The gelatin NPs, which degrade in response to matrix metalloproteinases overexpressed in tumors, allowed the NPs size to shrink from 188.2 to 55.9 nm, greatly improving tumor permeability and drug delivery efficiency.^164^

In a related study, AuNPs were functionalized with the EGFR‐targeting peptide GE11 (YHWYGYTPQNVI) and loaded with the photosensitizer Pc4 for targeted delivery to EGFR‐overexpressing GBM cells. These PEGylated AuNPs exhibited minimal dark toxicity and significant phototoxicity. Although NPs internalization was limited, Pc4 uptake depended on GE11 binding, likely due to enhanced surface interactions facilitating Pc4 desorption and cellular uptake. Using GE11, a peptide with high selectivity for EGFR and a size compatible with crossing the BBB, highlights its potential as an effective targeting agent.^165^

AuNPs hold significant potential for gene therapy, particularly in enabling targeted gene silencing for the treatment of brain tumors. A dendrimer‐entrapped AuNPs (Au DENPs) were successfully used as a platform for the delivery of VEGF or B‐cell lymphoma/leukemia 2 protein (BCL‐2) siRNA into a human glioma cell line.^166^ In another preclinical study, an RNA interference platform using spherical nucleic acid gold nanoconjugates was employed to regulate Bcl2‐L12 oncogene expression in GBM multiforme. After intravenous administration, the gold‐based nanoplatform successfully crossed the BBB, leading to a 26% reduction in Bcl2‐L12 gene expression and a 40% decrease in its protein levels in glioma cells.^167^ This gene silencing inhibited tumor growth and extended the survival of the mice. Additionally, AuNPs coated with Bcl2‐L12‐specific siRNA (NU‐0129) are currently in early‐stage clinical trials for intravenous injection to treat recurrent GBM.^168^

Recent studies demonstrated that AuNPs can improve the efficacy of RT by enhancing radiation absorption and generating localized heat for PTT. This combination approach showed potential in overcoming treatment resistance, particularly in aggressive tumors like GBM, by integrating the synergistic effects of both therapies. In a study by Guerra et al., the radio‐sensitizing effects of AuNPs and iron oxide NPs (SPION‐DX) were evaluated in human GBM cells (U87 and M059J) irradiated by 6 MV photons. The results indicated that AuNPs significantly enhanced radiosensitivity, especially in U87 cells, where they achieved a sensitization enhancement ratio (SER10%) of up to 2.04. In contrast, SPION‐DX demonstrated a smaller effect, with a maximum SER10% of 1.61. This suggests AuNPs are more effective in enhancing RT in GBM, particularly in radioresistant cell lines.^169^

## 5 AUNPS APPLICATION IN BRAIN CANCER TREATMENTS

The current treatment approach for brain tumors typically involves the surgical resection of the tumor, followed by RT and adjuvant chemotherapy.^170^ The presence of poorly defined tumor boundaries and the invasive nature of glioma cells within critical neurological areas pose significant challenges to achieving a comprehensive removal of the tumor tissue.^171^ Moreover, RT faces significant limitations in brain cancer treatment due to the hypoxic conditions within tumors and the reduced sensitivity of brain cancer cells to X‐radiation, leading to limited therapeutic effectiveness driven by cellular radio resistance. On the other hand, chemotherapy, which relies on small molecules to inhibit tumor growth, encounters several challenges, including poor physiological stability, lack of targeting specificity, and, most notably, the restrictive nature of the BBB.^172^ In fact, while the BBB protects the brain by preventing harmful substances from entering, it also obstructs the delivery of therapeutic agents, complicating the treatment of brain disorders.^154^ Thus, the challenges associated with conventional brain tumor therapies often result in minimal outcomes, with treatment efficacy remaining limited. In recent years, however, nanomedicine emerged as a promising tool for improving therapeutic results,^173^, ^174^, ^175^ particularly through the use of AuNPs,^176^, ^177^ which are increasingly recognized for their unique properties, such as enabling targeted drug delivery, enhancing stability and biocompatibility, allowing for controlled release, and improving penetration into tumor tissues.^178^

Table 2 presents some of the most recent studies on the use of AuNPs both in chemotherapy, more generally as drug delivery systems, and in RT to enhance treatment efficiency.

**TABLE 2:** Summary of studies investigating the application of AuNPs in chemotherapy as drug delivery systems, and in RT.

Application	Nanoparticles	In vivo/in vitro Model	Active component	Targeting component	Highlights	Ref
Drug delivery	PEG‐AuNPs	Mice bearing intracranial MDA‐MB‐231‐Br xenografts	DOX	HIV‐derived Trans‐Activating Transcriptional (TAT) activator peptide	Drug delivery of Dox for the treatment of brain metastatic breast cancer	[179]
Drug delivery	Au@Pt NPs	HEK‐293 U87‐MG D54 U251	Quinazoline	**—**	The nano system did not affect the proliferation of the control cell line, but was toxic towards GBM cells	[180]
Drug delivery	Au‐DOX	Orthotopic U87 tumor mouse	DOX	**—**	Inhibition of tumor growth and prolonged survival, with no adverse effects. Clearance of nanoparticles within 24 h.	[181]
Drug delivery	TMZ@AuNPs	C6 T98G Orthotopic glioma‐bearing rats	TMZ	Anti‐EphA3 antibody	Bypass the BBB and target glioma cells, enhancing the effectiveness of TMZ while minimizing peripheral toxicity and overcoming drug resistance.	[182]
Drug delivery	Hybrid AuNPs	U87 malignant glial cells	TMZ GC Decitabine	**—**	Overcoming glioma cell resistance and enhancing therapeutic outcomes through the synergistic action of the three drugs	[162]
Drug delivery	AuNPs	In vitro and in vivo GBM cells	DOX through hydrazone	Angiopep‐2	Improved drug delivery efficiency compared to free DOX	[163]
Drug delivery	Gelatin AuNPs		DOX	RGD Octarginine	Particles with smaller size showed better penetration ability, while RRGD modification could further improve permeability. In vivo, particles displayed the best glioma targeting and accumulation efficiency, with good colocalization with neo vessels.	[164]
TMZ RT	Hybrid AuNPs encapsulated with insulin	Orthotopic U87 tumor mouse	CTX	**—**	Suppression of tumor growth and extension of survival time. Decreasing tumor cell proliferation and repair mechanisms, while also targeting and elimination EGFR‐positive tumor cells.	[183]
RT	AuNPs‐ALA		**—**	**—**	Evaluation of the optimal neuroprotective dose of AuNPs against radiation‐injured male Wistar rat.	[184]
RT	d‐iGSNPs	Simulated BBB Orthotopic GL261 GBM mouse model	**—**	iRGD	Enhancement of the delivery and effectiveness of RT for GBM treatment.	[185]
RT	AuNPs@PEG	Human glioma U87 cells HBMECs		**—**	Radio sensitization enhancement ratio higher than that of the AuNPs group, significantly improving the X‐ray‐induced cytotoxicity against the tumor cells.	[186]
RT	GA‐GNPs	U251 cells	Gallic acid	**—**	Enhancement of cell survival outcomes compared to radiation treatment alone, particularly at higher concentrations of GA‐GNPs, revealing this combination treatment radio sensitizer towards GBM cells.	[187]

Abbreviations: AuNPs, gold nanoparticles; Au@Pt NPs, Au core encapsulated by platinum (PT) NPs; AuNPs‐ALA, AuNPs and alpha‐lipoic acid (ALA) combination; AuNPs@PEG, polyethylene glycol‐coated AuNPs; BBB, blood‐brain barrier; CTX, cetuximab; DOX, doxorubicin; GA‐GNPs, gallic acid‐loaded AuNPs; GBM, glioblastoma; GC, gemcitabine; PEG‐AuNPs, PEGylated AuNPs; RGD, Arg‐Gly‐Asp; RRGD, octaarginine–Arg‐Gly‐Asp; RT, radiotherapy; TMZ, temozolomide.

### 5.1 AuNPs in chemotherapy

Chemotherapy is a cancer treatment modality that involves the administration of pharmacological agents to eradicate malignant cells, inhibit their proliferation, and prevent metastasis.^188^ Despite its efficacy, chemotherapy affects healthy tissues and can significantly impact the patient's quality of life. This is due several factors, that is, the systemic distribution of the drugs throughout the body, the reduced specificity for tumor cells leading to unintended damage to normal tissues,^27^ the GBM cancer stem cell resistance, and the high heterogeneity of glioma cells.^189^ Moreover, the ability of BBB to selectively control the passage of substances, presents a significant challenge in the advancement of effective therapies for brain tumors. Many chemotherapeutic agents, such as cisplatin, paclitaxel, irinotecan, etc., exhibit limited capacity to cross this barrier, thereby reducing their therapeutic efficacy.^190^

Recent advances in nanotechnology enabled the development of innovative strategies to address challenges in chemotherapy, particularly in overcoming the BBB. NPs emerged as promising vehicles for the targeted delivery of chemotherapeutic agents, allowing for more precise treatment and minimization of systemic side effects.^191^ Among the various types of NPs, AuNPs stand out for their potential in enhancing drug delivery to tumor sites by facilitating the crossing of the BBB, thus ensuring higher concentrations of therapeutic agents at the target site.^192^ Thanks to their straightforward synthesis and the ease with which they can be functionalized with therapeutic agents, alongside their biocompatibility, AuNPs are particularly well‐suited for overcoming the BBB.^193^, ^194^

The biodistribution of AuNPs can be significantly influenced by their physio‐chemical characteristics, such as size, shape, surface coating, and surface charge. Specifically, AuNPs with a size of approximately 10 nm are able to cross the BBB^195^ relatively easily via passive diffusion,^196^ thus avoiding the obstructions encountered during traditional chemotherapy. However, in regions near tumors, where the BBB is often compromised (a phenomenon referred to as the blood‐brain tumor barrier), larger AuNPs, around 100 nm in size, may also pass through.

Beyond permeability, size plays a critical role in the uptake and clearance of NPs. Smaller AuNPs (<10 nm) tend to exhibit higher renal clearance, leading to faster elimination from the body, which may reduce drug accumulation at the tumor site. Conversely, larger AuNPs (50‐100 nm) experience slower clearance due to uptake by the RES, potentially prolonging circulation time but increasing liver and spleen accumulation.^197^

Surface charge is another key factor in drug delivery efficacy. Positively charged AuNPs typically exhibit greater cellular uptake due to electrostatic interactions with negatively charged cell membranes.^198^

To prevent rapid clearance from the bloodstream by the RES, the surface of AuNPs is typically modified with hydrophilic molecules such as PEG.^141^, ^155^

Morshed et al.^179^ developed a cell‐penetrating AuNPs‐platform aimed at enhancing drug delivery of Dox for the treatment of brain metastatic breast cancer, an aggressive malignancy affecting the CNS. In their work, they designed PEGylated AuNPs conjugated to HIV‐derived trans‐activating transcriptional (TAT) activator peptide (sequence: YGRKKRRQRRR) as well as Dox (TAT‐Au‐Dox) and injected by tail vein at a concentration of 3 mg/kg in mice bearing intracranial MDA‐MB‐231‐Br xenografts. The data demonstrated that 4 nm‐TAT‐Au‐Dox increased the average concentration of Dox in cells, as well as the number of Dox‐positive cells within a given population, even after exposure to lower drug doses. This suggested that the conjugation of Dox with NPs delivered overall higher doses to the cells, resulting in a greater therapeutic effect compared to Dox alone.

In another study, Stavropoulou et al.^180^ engineered a novel drug delivery nano system comprising bimetallic NPs, with a Au core encapsulated by platinum (Pt) NPs (Au@Pt NPs), which were further functionalized with a quinazoline‐based small molecule (Au@Pt@Q NPs), creating a theragnostic agent specifically targeted for the treatment of GBM. The cytotoxicity of the NPs was assessed using the MTT assay with a control human cell line, HEK‐293 (Human Embryonic Kidney cells), as well as three distinct human GBM cell lines: U87‐MG (wild type), D54 (wild type), and U251 (mutant). The viability profiles of these cell lines were compared following treatment with the synthesized nanostructures. Cytological analysis revealed that the NPs did not affect the proliferation of the control HEK‐293 cell line, but were toxic to U87‐MG, U251, and D54 GBM cells, thereby demonstrating selective toxicity towards GBM cells and highlighting their potential as targeted therapeutic agents for GBM.

Kruse et al.^181^ synthesized and functionalized 1.5 nm AuNPs with Dox (AuDox) and AlexaFluor647 (AuAF647) to investigate their potential for treating GBM in an orthotopic U87 tumor mouse model. Characterization revealed the NPs uniform size and high mobility, facilitating efficient cellular uptake in human cancer cell lines, including HeLa, T98‐G, brain endothelial cells, and astrocytes. In vivo, intravenously administered AuAF647 NPs successfully crossed the BBB, with approximately 23% accumulation in the brain tumor. The AuDox NPs effectively inhibited tumor growth and prolonged survival in the mice, with no observed adverse effects. Additionally, 84% of the NPs were excreted within 24 h, indicating their high systemic clearance and mobility.

A further study of Wang et al.^182^ focused on the development of TMZ‐conjugated 46 nm AuNPs functionalized with an antibody against the ephrin type‐A receptor 3 (anti‐EphA3‐TMZ@GNPs) for targeted GBM therapy via intranasal administration. AuNPs were designed to bypass the BBB and selectively target glioma cells, thereby enhancing the effectiveness of TMZ while minimizing peripheral toxicity and overcoming drug resistance. In vitro, the study was conducted on C6 rat glioma cells and T98G human GBM cells. The results showed a significant increase in cellular uptake and cytotoxicity in C6 cells, with an apoptosis rate of 54.9% compared to 14.1% with free TMZ. In TMZ‐resistant T98G cells, the IC_50_ of anti‐EphA3‐TMZ@GNPs was 18.5 times lower than that of free TMZ. Western blot analysis revealed a notable reduction in the expression of MGMT, a DNA repair enzyme responsible for TMZ resistance. The downregulation of MGMT enhanced the sensitivity of T98G cells to TMZ, suggesting that the AuNPs formulation could overcome resistance mechanisms. In vivo, experiments were performed on Fischer 344 rats implanted with C6 glioma cells to evaluate the therapeutic efficacy of the NPs. The results showed that the anti‐EphA3‐TMZ@GNPs extended the median survival to 42 days, significantly longer than the survival observed with free TMZ treatment.

All these results highlighted the potential of AuNPs as effective carriers for chemotherapeutic agents. Their properties, such as efficient drug delivery, targeted release, and ability to cross the BBB, make them promising candidates for enhancing the efficacy of chemotherapy, particularly in the treatment of challenging cancers like GBM. The ability of AuNPs to improve therapeutic outcomes while reducing systemic toxicity, positions them as a valuable tool in the development of more effective, targeted cancer therapies.

### 5.2 AuNPs in RT

RT induces cancer cell death by utilizing ionizing radiation, which, over time, became increasingly precise in generating irreversible DNA double‐strand breaks within tumor cells.^199^ This targeted approach allows for a more accurate delivery of X‐ray beams to the tumor, thereby minimizing the exposure of surrounding healthy tissue. However, despite these advancements in precision, a proportion of normal cells in proximity to the tumor inevitably receive radiation, leading to both acute and long‐term treatment‐related side effects.^200^

A major limitation of RT is its acute and long‐term toxicity, particularly affecting the CNS in pediatric brain tumor patients. Cognitive impairments, including reductions in intelligence quotient (IQ) and other functional losses, are well‐documented side effects. The key challenge, therefore, lies in improving local tumor control while minimizing toxicity, which can be achieved through highly accurate target delineation and the avoidance of radiation exposure to surrounding healthy tissues.^201^ To improve the efficacy of RT while minimizing associated side effects, radiosensitizers, that is, metallic NPs, gained significant attention in clinical research. These agents, in particular AuNPs, enhance tumor control and increase the likelihood of a cure by boosting the sensitivity of tumor cells to X‐rays when used in combination with RT.

Due to the high atomic number of Au, the physical sensitization mechanism in cancer cells involves the absorption and deposition of X‐ray energy by AuNPs, which then release electrons with energies ranging from eV to several keV. These electrons transfer their energy to the surrounding environment, primarily by generating ROS. Among these, photoelectrons and Compton electrons released by the NPs interact directly with cellular DNA, causing damage. Meanwhile, Auger electrons produced by the AuNPs react with water molecules to generate additional ROS which damage DNA and other cellular components, generate oxidative stress, affect cell cycle and cause bystander effect,^202^ leading to the apoptosis of cancer cells.^203^

Similarly, gadolinium‐based radiosensitisers exploit their high atomic number (*Z* = 64) to enhance radiation absorption, leading to increased production of low‐energy electrons that amplify DNA damage and ROS generation. Unlike AuNPs, gadolinium‐based NPs offer the added advantage of acting as contrast agents for MRI, providing both therapeutic and diagnostic benefits.^204^ Studies have also shown that combining gadolinium‐based NPs with RT results in increased apoptosis of tumor cells and prolonged survival in preclinical models. However, while gadolinium NPs benefit from MRI compatibility, AuNPs generally exhibit stronger X‐ray absorption properties due to their higher atomic number, which makes them particularly effective in amplifying the localized effects of radiation in tumor tissues.^205^

A critical factor influencing RT efficacy is tumor hypoxia, a condition characterized by low oxygen levels in the tumor microenvironment. Hypoxia significantly reduces the generation of ROS, as oxygen plays a key role in stabilizing and amplifying radiation‐induced DNA damage. This results in an overall decrease in RT effectiveness, leading to treatment resistance.^206^ Notably, AuNPs may help mitigate hypoxia‐induced resistance by locally increasing radiation dose deposition and enhancing the production of secondary electrons and ROS, thereby partially overcoming the protective effects of hypoxia.^207^

Gal et al.^183^ engineered a novel therapeutic approach by formulating a hybrid drug composed of radio‐sensitizing 20 nm AuNPs encapsulated with insulin. This system was designed to traverse the BBB and deliver tumor‐targeting antibodies, specifically cetuximab (CTX) (CTX‐INS‐GNPs), to brain tissue. Following intravenous administration in a mouse model of orthotopic GBM, established with human U87 cells, the AuNPs specifically localized within the tumor. This NPs delivery system was combined with TMZ and standard RT protocols. On day 14 post‐tumor implantation, mice bearing orthotopic GBMs were randomly assigned to different treatment groups. One group received the standard therapeutic regimen of TMZ and RT, which involved intraperitoneal TMZ administration (10 mg/kg for 5 consecutive days) and fractionated whole‐brain irradiation using 6 MV X‐rays (10 Gy delivered over 5 days, with 2 Gy per day); another group was treated with the same TMZ and RT regimen, but in combination with the CTX‐INS‐GNPs (containing 0.006 g AuNP and 3.7 mg/kg CTX per 200 µL injection), and a third group remained untreated as a control. The synergistic combination of functionalized AuNPs with conventional RT and TMZ resulted in a significant suppression of tumor growth and an extension of survival time. Histopathological examination further demonstrated that this combination treatment effectively decreased tumor cell proliferation and repair mechanisms, while also targeting and eliminating EGFR‐positive tumor cells.

To evaluate the neuroprotective effect of functionalized AuNPs from ionizing radiation damage, Abdelkader et al.^184^ investigated the effects of AuNPs and alpha‐lipoic acid (ALA) combination (AuNPs‐ALA) against radiation‐induced injury in male Wistar rats. Initially, three different doses of AuNPs alone (500, 1000, and 1500 µg/kg) were administered orally, 1 h before exposure to a single 7 Gy dose of gamma radiation. To assess the protective effects, biomarkers of oxidative stress (malondialdehyde (MDA); glutathione peroxidase (GPX)), DNA fragmentation, and histopathological alterations in the cortical and hippocampal regions of the brain were evaluated in both normal and irradiated rats. The optimal neuroprotective dose of AuNPs (1000 µg/kg) was then combined with ALA (100 mg/kg) to form the AuNPs‐ALA mixture, and its protective efficacy was compared to valproic acid, which served as a reference drug. All treatments were administered orally 1 h before the radiation exposure, and rats were assessed the following day using the same protocols as in the initial experiment. The AuNPs‐ALA combination demonstrated superior protective effects compared to either AuNPs or ALA alone, as it effectively reduced oxidative stress, DNA damage, and histopathological alterations. The combination therapy led to the normalization of MDA levels, enhanced GPX activity, and a restoration of DNA integrity in the cortex.

In another innovative work by Dong et al.,^185^ a novel therapeutic platform, termed d‐iGSNPs, was developed. This system comprised sub‐nanometer AuNPs (GSNPs), a BBB penetrating peptide (iRGD), and the cell cycle modulator α‐difluoromethylornithine. The primary aim of this study was to enhance the delivery and effectiveness of RT for GBM treatment. The researchers assessed the performance of d‐iGSNPs in both a simulated BBB model and an orthotopic GL261 GBM mouse model. Their findings demonstrated that d‐iGSNPs not only facilitated efficient BBB penetration but also improved the targeting of the tumor site. Furthermore, d‐iGSNPs sensitized GBM cells to RT, remarkably, when combined with a low‐dose RT regimen (2 Gy), the therapeutic effect was found to be nearly identical to that achieved with a higher radiation dose (4 Gy), suggesting that this approach could potentially reduce the adverse effects typically associated with high‐dose RT.

Another novel strategy for radio sensitizing glioma cells was developed by Wang et al.,^186^ using PEG AuNPs (AuNPs@PEG). This approach was tested both in vitro using human glioma U87 cells and human brain microvascular endothelial cells, as well as in vivo through subcutaneous injection into the left forelimb of female Balb/c mice. In vitro results following X‐ray irradiation demonstrated that AuNPs@PEG achieved a radio sensitization enhancement ratio (SER) of 1.74, 43.80% higher than that of the AuNPs group, significantly improving the X‐ray‐induced cytotoxicity against the tumor cells. In vivo experiments revealed that the combination of AuNPs@PEG and X‐ray therapy effectively reduced the expression of tumor‐associated molecules of 72.6% respect than PEG or AuNPs alone, while also inhibiting key processes involved in tumor progression, including invasion, proliferation, and migration.

A further strategy was performed by Jing et al.^187^ that developed gallic acid‐loaded AuNPs (GA‐GNPs) by conjugating gallic acid, a known antioxidant with anticancer properties, to 20 nm AuNPs. These GA‐GNPs were tested at varying concentrations (100, 150, and 200 μg/mL) on U251 GBM multiforme cells. The cells were then exposed to different doses of X‐ray irradiation (6 MV) ranging from 0 to 12 Gy. The results demonstrated that the combination of GA‐GNPs and radiation significantly enhanced cell survival outcomes compared to radiation treatment alone, particularly at higher concentrations of GA‐GNPs (150 and 200 μg/mL). This combination treatment effectively sensitized glioma U251 cells to radiation, highlighting the potential of GA‐GNPs as a promising radio‐sensitizing agent in GBM therapy.

## 6 AUNPS TOXICITY

The application of NPs in the biomedical field became a pivotal area of scientific investigation, particularly in the treatment of a wide range of diseases, including cancer. However, it is essential not only to evaluate their therapeutic advantages, such as the enhancement of treatment efficacy in chemotherapy or RT using AuNPs, but also to carefully consider their potential adverse effects, particularly with regard to toxicity, thus it is crucial to investigate the factors that affect it. It is precisely the inherent characteristics of AuNPs, that is, shape, size, surface chemistry, targeting ligands, elasticity, and composition, that significantly influence their toxicity profiles.^208^ As an example, studies examined the biodistribution and toxicity of AuNPs in various shapes, particularly nanorods and nanoshells. Nanorods are typically synthesized with cetrimonium bromide (CTAB), a cationic surfactant that influences their shape, but CTAB found to be toxic to cultured cells in vitro.^209^ In contrast, nanoshells exhibited dose‐dependent toxicity when tested on African green monkey kidney (Vero) cells,^210^ indicating that their toxic effects may vary with concentration.^211^

A few mouse model studies reported varying toxicity of AuNPs depending on size. Zhang et al.^212^ investigated the in vivo toxicity of PEG‐coated AuNPs with sizes of 5, 10, 30, and 60 nm in male mice. The mice were administered 200 µL of NPs solution at a dosage of 4000 µg/kg. Biochemical analysis revealed that both the 10 and 60 nm PEG‐coated AuNPs led to a significant increase in alanine transaminase (ALT) and aspartate transaminase (AST) levels, indicating liver damage. Biodistribution studies demonstrated that the 5 and 10 nm NPs primarily accumulated in the liver, while the 30 nm particles were more prevalent in the spleen. Additionally, the 5, 10, 30, and 60 nm particles were detected in the blood and bone marrow, with the 5 and 60 nm particles showing a particular propensity to aggregate in blood cells. In another investigation conducted by Pan et al.,^213^ water‐soluble AuNPs stabilized by triphenylphosphine derivatives, with sizes ranging from 0.8 to 15 nm, were studied for their cytotoxic effects. The NPs were tested on four distinct cell lines representing key functional cell types with barrier and phagocytic properties: connective tissue fibroblasts, epithelial cells, macrophages, and melanoma cells. The study found that the 1.4 nm AuNPs were the most cytotoxic, with IC_50_ values ranging from 30 to 56 μM, depending on the specific AuNP‐cell line combination. In contrast, larger particles (15 nm) and Tauredon (gold thiomalate) exhibited no toxicity even at concentrations 60 times and 100 times higher, respectively. The cellular response to these NPs was size‐dependent: the 1.4 nm particles induced rapid necrotic cell death within 12 h, whereas the closely related 1.2 nm particles primarily triggered programmed cell death through apoptosis.

While various studies reported shape and size‐dependent toxicities of AuNPs, direct comparison remains difficult due to the variability in experimental conditions, such as NPs size, coating agents, concentrations, and exposure durations, all of which can significantly influence the observed outcomes.^214^

Additionally, beyond acute toxicity, the long‐term in vivo effects of AuNPs require further investigation, particularly regarding immunogenicity and off‐target accumulation in organs such as the liver and spleen, raising concerns about potential chronic toxicity and inflammatory responses.^215^ The immune system may recognize certain formulations of AuNPs as foreign, triggering unwanted immune activation or, conversely, immune suppression, which could impact their therapeutic applications. The extent of this response depends on multiple factors, including NPs size, surface chemistry, and the presence of specific biomolecular coronas formed upon interaction with plasma proteins.^216^ Moreover, the clearance mechanisms of AuNPs remain an essential factor in determining their long‐term biological effects.^217^

With regard to the composition of AuNPs and surface chemistry, it is clear that, depending on the synthesis method used, these factors vary greatly, affecting the safety of nanomaterials. Connor et al.^218^ demonstrated how CTAB used with other chemicals (cysteine, citrate, glucose, etc.) during the synthesis process did not exhibit cytotoxic action in human leukemic cells, thing that happens when it is used alone. In the same way, Alkilany et al.^219^ evaluated the toxicological assays of CTAB‐capped nanorod solutions with human colon carcinoma cells (HT‐29), revealing that the apparent cytotoxicity is caused by free CTAB in solution.

In conclusion, while the toxicity of AuNPs has been explored in a number of studies, the available literature remains limited and highly heterogeneous. Variations in the results are largely attributable to differences in the type of NPs used, their synthesis methods, and their chemical composition. Factors such as size, surface charge, coating materials, and the presence of targeting ligands can all significantly influence the biological interactions and toxicity profiles of AuNPs. Moreover, discrepancies in experimental protocols, including variations in dose, exposure time, and the cell or animal models employed, further complicate direct comparisons across studies. As such, while AuNPs hold great promise for biomedical applications, a more consistent and standardized approach in toxicity testing is essential for a clearer understanding of their safety and therapeutic potential.

## 7 CONCLUSIONS AND FUTURE PERSPECTIVES

Nanotechnology is reshaping the landscape of cancer treatment, offering innovative solutions to enhance the efficacy of traditional therapies. Among NPs, AuNPs emerged as exceptional tools in the fight against aggressive brain tumors like GBM. Their unique physical and chemical properties, including a high atomic number, biocompatibility, and plasmonic characteristics, position them as powerful allies in targeted cancer therapy.

Conventional methods such as surgery, chemotherapy, and RT remain critical for managing brain cancer, yet the prognosis for full recovery often remains grim, underscoring an urgent need for advanced therapeutic strategies. AuNPs act as radiosensitizers in RT, selectively accumulating in tumor cells to amplify radiation effectiveness while sparing healthy tissues. Their surface can be functionalized for precision drug delivery in chemotherapy, reducing off‐target toxicity and enhancing treatment outcomes. Beyond these applications, AuNPs are increasingly being integrated with emerging therapies, such as immunotherapy and chimeric antigen receptor‐T cell treatments, to further enhance their efficiency. AuNPs can serve as a platform for delivering immune‐stimulating agents, enabling synergistic interactions that target both tumor cells and the immunosuppressive microenvironment of brain cancer.

Despite these promising developments, significant challenges remain. Long‐term toxicity, regulatory hurdles, scalability, and the optimization of ligand functionalization for precise targeting must still be addressed to facilitate clinical translation. Nevertheless, the future of AuNPs in cancer therapy is promising, with ongoing in vitro and in vivo research driving innovation. The convergence of AuNPs with cutting‐edge approaches such as nanomedicine‐based immunotherapies and gene‐editing technologies offers transformative potential. As these advancements progress, the integration of AuNPs into a multidisciplinary framework of cancer treatment may pave the way for novel, effective, and personalized strategies, bringing new hope to patients battling brain cancer.

## AUTHOR CONTRIBUTIONS

Simona Tarantino and Valeria De Matteis conceived the idea of manuscript; Simona Tarantino, Annalisa Bianco, Valeria De Matteis, Edoardo Scarpa, and Rosaria Rinaldi wrote and edited the manuscript.

## CONFLICT OF INTEREST STATEMENT

The authors declare no conflicts of interest.

## ETHICS STATEMENT

Not applicable.

## Data Availability

The manuscript does not contain novel data, and all the information provided can be sourced from literature.
